# Is the Duration of Diabetes Diseases Positively Associated With Knowledge About Diabetic Complications? Knowledge of Diabetes Mellitus Complications and Associated Factors Among Type-2 Diabetic Patients in Public Hospitals of Addis Ababa, 2020

**DOI:** 10.3389/fpubh.2021.812586

**Published:** 2022-02-21

**Authors:** Getachew Zewdu Chekol, Daniel Mengistu, Addisu Waleligne Tadesse

**Affiliations:** ^1^Public of Health Emergency Management, Aletawondo Woreda Health Office, Aleta Wondo, Ethiopia; ^2^School of Nursing and Midwifery, College of Health Science, Addis Ababa University, Addis Ababa, Ethiopia; ^3^Department of Public Health, College of Health Science, Salale University, Fiche, Ethiopia

**Keywords:** Addis Ababa, diabetic complication, DM patients, Ethiopia, knowledge

## Abstract

**Background:**

Although the importance of educational programs in the prevention and control of diabetes mellitus (DM) and its complication is well-recognized, there are concerns about whether these programs are achieving the desired goal of increasing knowledge of DM and its complication in developing countries. Therefore, this study assessed knowledge of DM complications and associated factors among type-2 diabetic patients in public hospitals of Addis Ababa.

**Method:**

Simple random sampling technique was used to select 422 participants. Data were entered to EpiData Version 4.6.0.1 and analyzed using SPSS Version 25 software. Multicollinearity and model goodness-of-fit was checked. A multivariate logistic regression model at 95% CI was used to identify the predictors.

**Result:**

The overall knowledge of diabetes complications among diabetic patients in the Hospitals of Addis Ababa was 54.9%. In the fitted model, being a profession of governmental workers [adjusted odds ratio (AOR) = 3.12, 95% CI (1.33, 7.34)] and merchants [AOR = 2.54, 95% CI (1.16, 5.56)]; DM duration 5–10 years [AOR = 0.41, 95% CI (0.23, 0.73)] and ≥10 years [AOR = 0.36, 95% CI (0.19, 0.69)]; family history of DM [AOR = 1.68, 95% CI (1.03, 2.75)]; and participating in diabetic counseling [AOR = 2.41, 95% CI (1.50, 3.86)] were significantly associated with knowledge about DM complications.

**Conclusion and Recommendation:**

The overall knowledge of diabetes complications among diabetic patients in the Hospitals of Addis Ababa was 54.9%. It was determined by the duration of DM, current profession, family history, and participation in diabetes counseling. Hence, emphasis on sustaining knowledge about diabetes complications for patients who are more than 5 years since diagnosis and attention is needed about knowledge diabetic farmers.

## Introduction

Diabetes mellitus (DM) is a metabolic disorder of multiple etiologies characterized by chronic hyperglycemia with disturbances of carbohydrate, protein, and fat metabolism resulting from defects in insulin secretion, action, or both (1–3). Among all diabetes cases, about 90–95% are type-2 and the remaining 5% are type-1 in which the cause is mostly idiopathic (3). Increased blood glucose is a usual manifestation of uncontrolled diabetes, through time it leads to serious damage to different organs and systems. For instance, the cardiovascular, excretory, sensory system, and among organs heart, blood vessels, eyes, kidneys, and nerves are affected by DM (4).

Diabetes mellitus is one of the fastest-growing and the most common chronic endocrine and metabolic disorders that affects millions of people, and it attributes to 8% of all-cause mortality in the world (5). Due to the severity of its complications, it comprises an important public health problem (1, 6). The highest proportion (73.1%) of death due to DM is recorded in Africa, though the region is the lowest both in prevalence and incidence of DM.

At present, many of the health systems in sub-Saharan Africa struggle to cope with infectious diseases. Meeting the goals of the UN high-level meeting on non-communicable diseases (to reduce premature mortality from None Communicable diseases (NCDs) by 25% by 2025) and Sustainable Development Goals (to reduce premature mortality from NCDs by a third by 2030) requires a coordinated approach within countries, which starts with a firm consideration of disease burden, needs, and priorities (7).

With the growing prevalence and increasing costs of treatments, diabetes is set to add further pressure on healthcare systems worldwide, particularly among countries at the forefront of economic development (8). Premature death and disability due to diabetes are associated with a negative economic impact on countries, often called the “indirect costs” of diabetes. These will have a devastating impact on the quality of life and the economic status of both individuals and the society they live in (3). The annual direct medical expense per patient varied among different types of complications and increased dramatically with the number of diabetic complications and patients were exposed to great financial risk (9). In 2015, the overall cost of diabetes in sub-Saharan Africa was US$ 19.45 billion or 1.2% of cumulative gross domestic product (GDP); around 55.6% of this cost arose from direct costs. The total cost is estimated to increase between $35.33 billion (1.1% of GDP) and $59.32 billion (1.8% of GDP) by 2030 (7).

This silent, but impending, public health problem would bring down challenges on the healthcare system and on the economy of most developing countries in the near future. This is because a significant proportion of individuals who suffer from the condition in these countries are within the reproductive age (5, 7). Rapid demographic, sociocultural, and economic transitions are driving increases in the risk and prevalence of diabetes in sub-Saharan Africa. The health systems have inadequacies at all levels to provide adequate management for diabetes and its associated risk factors and sequelae. There is a paucity in the availability of simple equipment for diagnosis and monitoring, a lack of sufficiently knowledgeable healthcare providers, insufficient availability of treatments, a dearth of locally appropriate guidelines, and few disease registries (7).

Low level of knowledge and/or awareness toward DM and its complication attributed to high prevalence and incidence of lower limb amputation, diabetic renal diseases, diabetic eye diseases, and acute complications of DM (10). It also leads to a social and long-term economic impact on individuals and society (3). Being literate, better income status, and having a family history of DM are identified as predictors for good awareness of DM complications of diabetes patients in a previous study (11, 12).

Even though service delivery for DM diagnosis and management is escalated, the burden of complications due to diabetes is increasing from time to time in Ethiopia (10, 13, 14). Due to the social burden and subsequently the economic implication as a result of the DM complications, a high level of awareness is needed among diabetic patients about these debilitating complications. Although the importance of educational programs in the prevention and control of DM and its complication are well-recognized, there are concerns about whether these programs are achieving the desired goal of increasing awareness of DM and its complication in developing countries.

Since there are no specialized centers for the management of DM and its complication in Sub-Saharan Africa (SSA) settings, patient education, and awareness creation become a central component in the prevention and control of this disease in SSA (15, 16). Although the importance of educational programs in the prevention and control of DM and its complication is well-recognized, there are concerns about whether these programs are achieving the desired goal of increasing knowledge of DM and its complication in developing countries. Therefore, the purpose of this study was to determine the level of knowledge about DM complications and identify factors associated with knowledge among patients with type-2 DM in the public hospitals of Addis Ababa.

The findings of this study, in general, will inform the health managers at each level and Healthcare worker (HCWs) about the level of knowledge of diabetic patients about DM complications. The findings will help the hospital managers and concerned stakeholders to redirect their action toward knowledge creation about DM complications. The finding of this study will be used as a reference for future researches.

## Methods and Materials

### Study Area and Study Period

This study was conducted from October 10 to November 10, 2020 in the public hospitals of Addis Ababa, Ethiopia. Addis Ababa is the capital city of Ethiopia with an area of 540 square km. The total population of the city is about 3.3 million with 5,046 people per kilometer. The Administrative region has structured with 10 sub-cities and 106 districts. According to Ethiopian Public Health Institute (EPHI), there are 13 home-to-home treatment services, 1,047 different private clinics, 104 health centers, 4 primary hospitals, 24 general hospitals, and 24 specialized hospitals and treatment centers *[Source: Ethiopian Public Health Institute (EPHI), 2020]*.

### Study Design

An institution-based cross-sectional study design was used to conduct the study.

### Study Population

All patients with type-2 DM at Public Hospitals of Addis Ababa were the source population. All type-2 diabetic patients who came to the selected diabetic clinics during the data collection period were the study populations.

### Inclusion Criteria and Exclusion Criteria

- All type-2 diabetic patients who were on medication for more than 1 year and who were ≥18 years old were included.
- All patients with type-2 DM who were seriously ill and health professionals were excluded.

### Sample Size and Sampling Procedures

#### Sample Size

- The sample size was determined as follows based on a single population proportion formula with a 95% level of confidence, a 5% marginal error, and *p* = 48.5% (proportion of knowledge about DM complication) from the previous study (11).

Sample size = n = $\frac{\frac{z_{\alpha }^{2}}{2}}{d^{2}}$ p(1-p)
= $\left(\frac{1.96^{2}}{0.05^{2\;}}\right)$ (0.485) (1 – 0.485)
= 384; considering 10% non-response rate, the final sample size by using a single population proportion formula is 422.

The sample size for factors was determined by using EPI INFO Version 7 Stat Calc by considering income, sex, and level of education (11) (Table 1). Hence, the sample size obtained by the proportion was higher than the sample size obtained by factors, the sample size for this study was 422.

**Table 1:** Calculated sample size for factors using two populations proportions by Open Epi version 7.

**Assumptions**	**Variables**		
	**Income**	**Level of education**	**Sex**
	• Two-sided CL = 95% • Power = 80% • Ratio of Unexposed to Exposed = 1 • % of unexposed with outcome = 31.6% • OR = 3.22	• Two-sided CL = 95% • Power = 80% • Ratio of Unexposed to Exposed = 1 • % of unexposed with outcome = 45.5% • OR = 3.793	• Two-sided CL = 95% • Power = 80% • Ratio of Unexposed to Exposed = 1 • % of unexposed with outcome = 36.4% • OR = 4.67
Calculated *n*	110	92	68
Final *n* (10% contingency)	121	101	75

### Sampling Procedures

**Figure 1:**
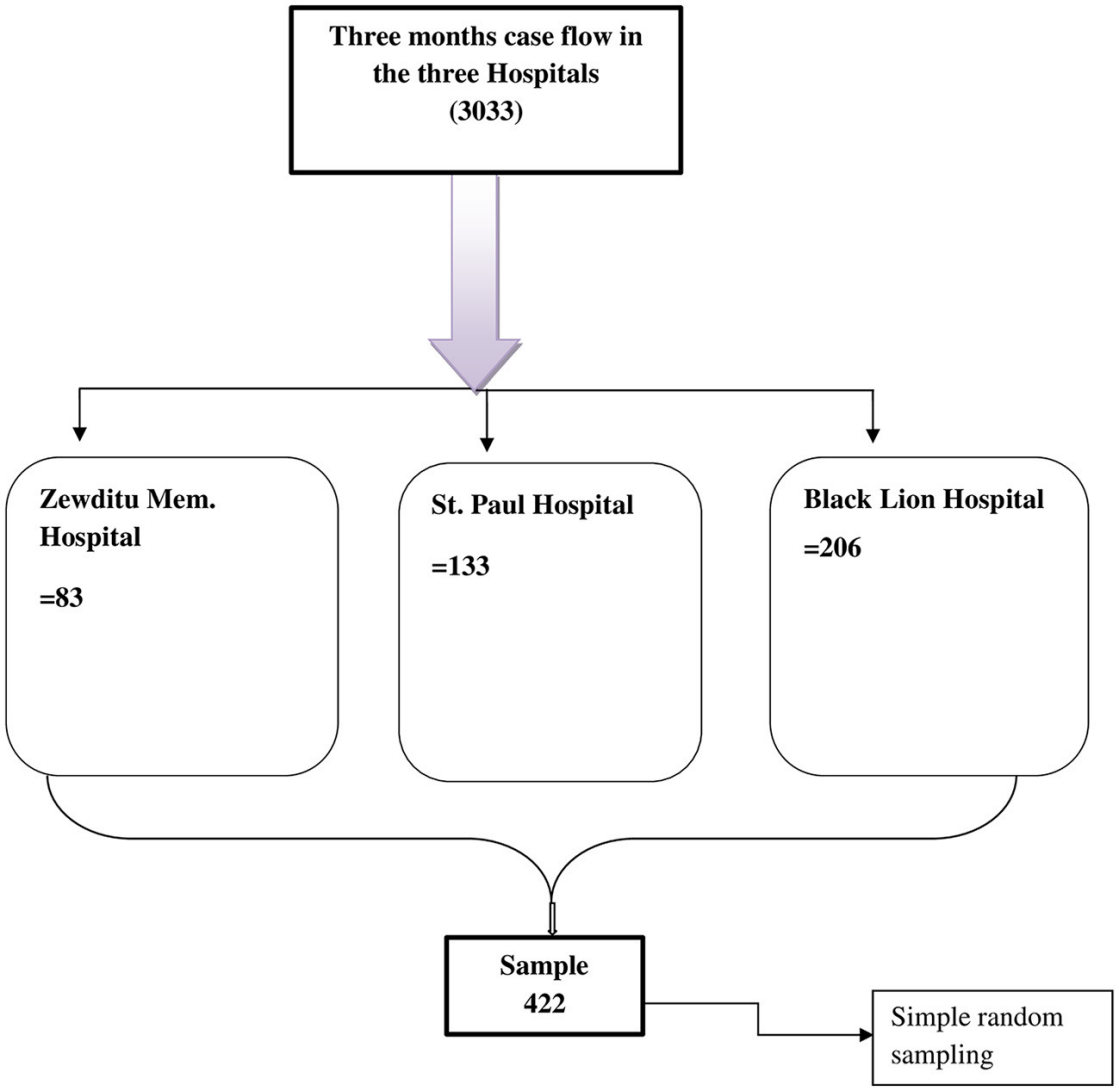
Schematic representation of the sampling procedure, 2020.

Proportional numbers of study participants were allocated to each hospital according to the number of diabetic patients on the follow-up in the last 3 months. Individual participants who fulfilled the inclusion criteria were selected by simple random sampling from each hospital. The sampling fraction used to allocate the sample size to each hospital was 0.14 (422/3,033) (Figure 1).

### Variables of the Study

The dependent variable was knowledge of DM complications. Independent variables were socio-demographic variables (age, sex, marital status, occupation, residence, income, family history of DM, and level of education), duration since diagnosis as diabetic, participation in diabetes counseling, source of information about DM complication, and existence of diabetic education program.

### Operational Definitions

- Diabetes mellitus complications: Presence of one or more of the following diabetes complications: retinopathy, diabetic foot, renal complication, stroke, heart complication, neuropathy, hypertension, tooth decay, and sexual dysfunction (11).
- Rural residence: Settling in the countryside outside of big cities or towns in Ethiopia is referred to as rural residents (11).
- Family history of DM: Having at least one first-degree relative with diabetes (11).
- Good knowledge: Knowledge score equal to or above the mean.

### Data Collection Procedures (Instrument, Personnel)

A pre-tested structured interviewer-administered questionnaire, which is adapted from different literature, was used to collect the data (10, 11, 17, 18). The questioner contains 32 diabetes complication-related knowledge items. The possible correct answers for assessing knowledge of diabetes complications were 32. Three diploma nurses and one supervisor were recruited as data collectors, and they were given 1-day training. Data collectors had visited all three hospitals and explained the aim of the research to the facility managers and study participants.

### Data Quality Control Analysis

The questionnaires were prepared first in English, then translated to the Amharic language, and then again back-translated to English. The pre-test was done on 5% of the sample at Tirunesh Beijing Hospital and modification of some questions was made before the main data collection. Data were checked daily for completeness and consistency. Data were coded manually, and to assure the quality of data during data entry, 10% of the data were re-entered into EpiData Version 4.6.0.0. Then data cleaning was performed by SPSS Version 21 software to check and correct inconsistencies and missing values. For this purpose, frequency and cross-tabs were utilized.

Univariate analyses such as measures of central tendency and measures of dispersion for continuous variables were computed. Frequency distribution was done for categorical data. Binary logistic regression analysis was done to each independent variable with outcome variable to select candidate variables with *p* < 0.25. Multicollinearity was checked by using variance inflation factor (VIF) at 10%. Then entered into multivariable analysis to identify factors associated with the outcome variable and control for confounders. Model fitness will be checked by the Hosmer and Lemeshow goodness of test. Backward stepwise logistic regression will be used to determine factors associated with *p* < 0.05 with their respective adjusted odds ratio (AOR) and 95% CI. Finally, reports were presented by the text, figures, and tables.

### Ethical Consideration

The study was approved by the Institutional Research Ethics Review Committee of Kea-med Medical College, which was written on October 2020 with ref. no of KMC/IRB/014/12. A formal letter was written to Addis Ababa Health Office and then a supporting letter was written to hospital administers. Written consent was taken as informed consent from each study participant.

## Results

### Socio-Demographic Characteristics of the Participants

A total of 377 participants were included in the study. The response rate was 89.3%. The remaining 10.7% of the participants were refused to participate. The majority (82.5%) of the participants were urban residence. The mean age was 51.9 years (SD ± 16 years). The age group ≥46 years covered the highest percentage (62.3%). More than half of (57.8%) participants were married (Table 2).

**Table 2:** Socio-demographic characteristics of study participants in public hospitals of Addis Ababa, Ethiopia, 2020 (*n* = 377).

**Characteristics**		**Frequency**	**Percentage**
Age	18–30	8	2.1
	31–45	134	35.5
	≥46	235	62.3
Sex	Male	167	44.3
	Female	210	55.7
Marital-status	Single	28	7.4
	Married	218	57.8
	Divorced	77	20.4
	Widowed	54	14.3
Educational level	Cannot write and read	50	13.3
	Primary	102	27.0
	Secondary and above	225	59.7
Current profession	Farmer	40	10.6
	Government worker	92	24.4
	Merchant	123	32.6
	House wife	109	28.9
	NGO	13	3.4
Residence	Rural	66	17.5
	Urban	311	82.5
Duration of DM	0–4	111	29.4
	5–10	157	41.6
	>10	109	28.9
Family history	Yes	242	64.2
	No	135	35.8
Income	<500	13	3.4
	500–1,500	88	23.3
	1,501–2,500	72	19.1
	>2,500	204	54.1
Sources of information	TV/radio	337	89.4
	Health worker	40	10.6
Participation in diabetic counseling	Yes	207	54.9
	No	170	45.1
Diabetic education	Yes	4	1.1
	No	373	98.9

### Knowledge Status on Diabetic Complication of Patients With Type-2 DM

**Figure 2:**
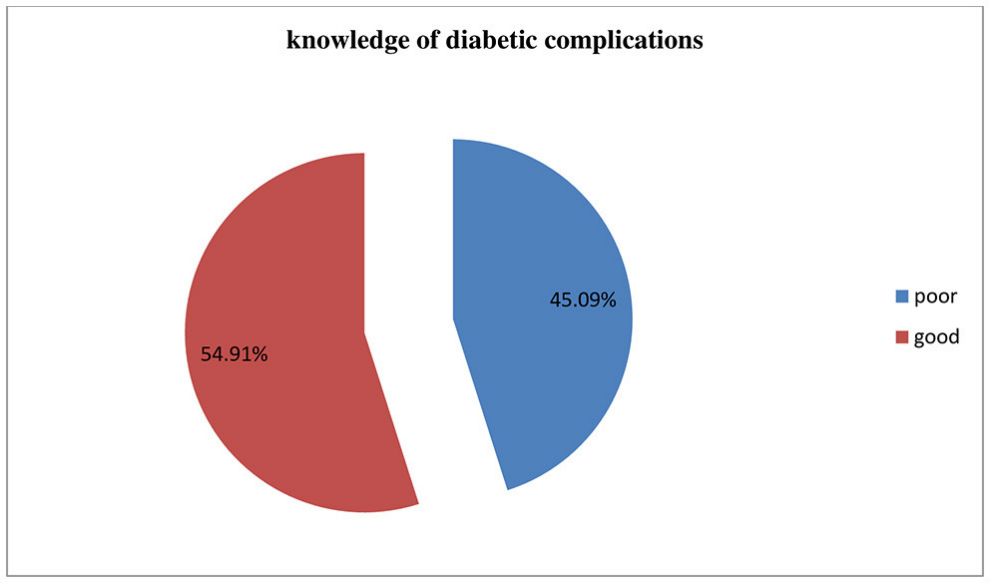
Knowledge on diabetes mellitus complications among Type-2 diabetic patients in public hospitals of Addis Ababa, 2020.

The study showed that the overall knowledge of DM complications among diabetic patients was 59.9% [95% CI (50.1, 60.7)]. A total of 207 (59.9%) diabetic patients had a knowledge score of the mean (22.2) and above (Figure 2).

Among the diabetic patients who participated in this study, only 64 (17%) of them knew that stroke could be one of the complications of DM. The majority of (82.8%) study participants were not aware that sexual dysfunction could be attributed to DM (Table 3).

**Table 3:** The distribution of diabetic patients by their knowledge of diabetic complications in public hospitals; Addis Ababa, Ethiopia, 2020.

**Knowledge items**		**Yes/true *n* (%)**	**No/false *n* (%)**	**I don't know *n* (%)**
What is the normal fasting blood sugar level?		121 (32.1)	256 (67.9)	0
What are the most common symptoms of high blood sugar?	Increased thirst	195 (51.7)	178 (47.2)	4 (1.1)
	Frequent urination	237 (62.9)	140 (37.1)	0
	Blurring of vision	323 (85.7)	54 (14.3)	0
	Weakness	306 (81.2)	71 (18.8)	0
	Dry mouth	229 (60.7)	148 (39.3)	0
	Confusion	109 (28.9)	268 (71.1)	0
What are the most common symptoms of low blood sugar?	Palpitation	229 (60.7)	148 (39.3)	0
	Tremor	291 (77.2)	86 (22.8)	0
	Sweating	322 (85.4)	54 (14.3)	1 (0.3)
	Blurring of vision	315 (83.6)	61 (16.2)	1 (0.3)
	Decreased coordination	84 (22.3)	292 (77.5)	1 (0.3)
Do you know that diabetes can cause complications in organs of our body?		374 (99.2)	3 (0.8)	0
What are common complications of organs?	Diabetic foot	266 (70.6)	109 (28.9)	2 (0.5)
	Eye complication	323 (85.7)	52 (13.8)	2 (0.5)
	Heart complication	259 (68.7)	118 (31.3)	0
	Neuropathy	163 (39.7)	214 (56.8)	0
	Renal complication	321 (85.1)	56 (14.9)	0
	Stroke	64 (16.9)	312 (82.8)	1 (0.3)
	Tooth decay	176 (46.7)	200 (53.0)	1 (0.3)
	Sexual dysfunction	63 (16.7)	310 (82.2)	4 (1.1)
Can dietary modification prevent diabetic complication		300 (79.6)	8 (2.1)	69 (18.3)
Is physical exercise help to prevent diabetic complication		324 (85.9)	12 (3.2)	41 (10.9)
If you are beginning to have a low blood glucose reaction, you should?	Exercise	356 (94.4)	21 (5.6)	0
	Lie down and rest	326 (86.5)	51 (13.5)	0
	Drink some juice	333 (88.3)	44 (11.7)	0
	Rapid-acting insulin	287 (76.1)	90 (23.9)	0
Is diabetic education existed in the facility where you have follow-up?		251 (66.5)	4 (1.1)	122 (32.4)
Knowledge status		Good		207 (54.9)
		Poor		170 (45.1)

### Factors Associated With Knowledge of Diabetic Complications

In identifying independent factors for good knowledge of diabetic complications, a statistical association was observed among diabetic patients who were governmental workers [AOR = 3.12, 95% CI (1.33, 7.34)] and merchants [AOR = 2.54, 95% CI (1.16, 5.56)] in comparison to farmers. Patients with DM of duration 5–10 years [AOR = 0.41, 95% CI (0.23, 0.73)] and ≥10 years [AOR = 0.36, 95% CI (0.19, 0.69)] were 60 and 64% less likely to have good knowledge of diabetic complications, respectively, compared to <5 years duration. Furthermore, patients who had a family history of DM [AOR = 1.68, 95% CI (1.03, 2.75)] were 1.6 times more likely to have good knowledge about DM complications. Patients who participated in diabetic counseling [AOR = 2.41, 95% CI (1.50, 3.86)] were 2.4 times more likely to have good knowledge about DM complications (Table 4).

**Table 4:** Bivariate and multivariable logistic regression analysis of knowledge of diabetes mellitus complications among Type-2 diabetic patients in public hospitals of Addis Ababa, 2020.

**Factors**	**Knowledge**		**COR 95% CI**	***P*-value**	**AOR 95% CI**
	**Good freq**.	**Poor freq**.			
**Age**					
≥46	107	128	0.84 (0.2, 3.42)	0.80	
31–45	96	38	2.56 (0.6, 10.62)		
18–30	4	4	1.00		
**Sex**					
Male	105	62	1.79 (1.18, 2.71)	0.006^*^	
Female	102	108	1.00		
**Educational status**					
Secondary and above	153	72	8.5 (4.02, 17.95)	0.000^*^	
Primary	44	58	3.03 (1.37, 6.73)	0.006^*^	
Can't write and write	10	40	1.00		
**Profession**					
NGO worker	10	3	5.56 (1.32, 23.45)	0.02^*^	3.13 (0.67, 14.65)
House wife	31	78	0.66 (0.31, 1.42)	0.29	0.58 (0.26, 1.3)
Merchant	81	42	3.21 (1.53, 6.74)	0.002^*^	2.54 (1.16, 5.56)^**^
Government worker	70	22	5.3 (2.38, 11.8)	0.000^*^	3.12 (1.32, 7.34)^**^
Farmer	15	25	1.00		
**Residence**					
Urban	179	132	1.84 (1.07, 3.15)	0.026^*^	0.87 (0.19, 3.86)
Rural	28	38	1.00		
**Duration since diagnosis of DM**					
≥10	43	66	0.22 (0.12, 0.39)	0.000^*^	0.36 (0.19, 0.69)^**^
5–10	81	76	0.36 (0.21, 0.61)	0.000^*^	0.41 (0.23, 0.73)^**^
<5	83	28	1.00		
**Family history**					
Yes	141	101	0.146 (0.96, 2.23)	0.08^*^	1.68 (1.03, 2.75)^**^
No	66	69	1.00		
**Income**					
>2,500	137	67	3.27 (1.03, 10.38)	0.044^*^	
1,501–2,500	40	32	2.00 (0.6, 6.71)	0.262	
501–1,500	25	63	0.63 (0.19, 2.13)	0.462	
<500	5	8	1.00		
**Source of information**					
Health worker	189	148	1.56 (0.81, 3.02)	0.185^*^	
TV/Radio	18	22	1.00		
**Participation in diabetic counseling**					
Yes	139	68	3.07 (2.01, 4.68)	0.000^*^	2.41 (1.5, 3.86)^**^
No	68	102	1.00		

* *Variables candidate for multivariate logistic regression*.

** *variables significantly associated with knowledge of DM complications at multivariate logistic regression using stepwise backward LR method*.

*freq., frequency; COR, crude odds ratio; AOR, adjusted odds ratio*.

Age, sex, educational status, marital status, residence, income, sources of information, and awareness of diabetic education in the health facility service were significantly associated with knowledge of diabetic complications during binary logistic regression analysis. However, they did not remain as predictors during multivariate logistic regression analyses.

## Discussion

### Knowledge of DM Complications

Knowledge of diabetic complications among patients with type-2 DM in the public hospitals of Addis Ababa was 54.9% [95% CI (49.6, 59.5)]. Duration of DM since diagnosis, current profession, family history, and participation in diabetic counseling were identified as predictors for good knowledge about DM complications.

In this study, the overall good knowledge of DM complications was relatively higher compared to the finding from Northwest Ethiopia (48.5%) (11). This little discrepancy might be due to population variation; 82.5% of study participants in this study were urban dwellers but only 60.4% of participants were urban-residential in Northwest Ethiopia. This higher number of living in urban, particularly in Addis Ababa, may raise the awareness/knowledge of patients with DM toward DM complications.

About 70.6% [95% CI (66, 75.1)] of study participants were aware of the diabetic foot as a complication of DM. This finding was in-line with the finding from Northwest Ethiopia (73.9%) (11), Nepal (72%) (18), Pakistan (72.9%) (19), Makah (71.2%) (20), and Nigeria (66.2%) (21).

About 85.7% [95% CI (82.4, 89.1)] of study participants were aware of diabetic eye complications, which is higher than findings from Northwest Ethiopia (72.6%) (11) and Felege-Hiwot Referral Hospital, Ethiopia (54.4%) (22). This might be due to the variation in the study population because the majority of study participants in this study are urban dwellers. However, this was in line with the studies conducted in Malaysia (86–87.2%) (23, 24), Turkey (88.1%) (12), and Myanmar (86%) (25).

Two-thirds (68.7%) [95% CI (63.7, 73.7)] of study participants were aware of heart-related complications attributed to DM, which was consistent with the findings from Nepal (68%) (18). Although it is not in line with the given CI, the finding is approximately similar to the findings from Northwest Ethiopia (63.2%) (11). However, this finding was higher than the findings from India (50%) (26), Bangladesh (48.9%) (27), Pakistan (50–60%) (19), Gambia (5.5%) (28), Ghana (9.2–35.4%) (29), and Nigeria (39.2%) (21). This difference might arise from time variation and/or methodological approach.

Only 16.7% [95% CI (13.2, 20.4)] of study participants were aware of impotency that (sexual dysfunction) could be a complication of DM. However, this finding was lower than findings from India (24%) (26), Makah (42.5%) (20), Nigeria (41.9%) (21), and Ghana (25.4%) (29). This might be due to socio-demographic variation and/or educational and counseling approach from healthcare workers to patients with DM.

Stroke was known only by 17% [95% CI (13.3, 20.8)] of study participants. This was consistent with the finding from Saud Arabia (18.7%) (20) and Bangladesh (15.2%) (27). However, it was lower than the findings from Northwest Ethiopia (39.1%) (11), Nepal (38%) (18), India (46.8%) (26), Pakistan (56.25%) (19), and Nigeria (41%) (21).

The majority (85.1%) [95% CI (81.6, 88.4)] of study participants had knowledge about diabetic renal complications. This finding was inconsistent with the finding from Northwest Ethiopia (53.7%) (11), India (53.2%) (26), Nepal (76%) (18), Bangladesh (13%) (27), Pakistan (66.6%) (19), Nigeria (54%) (21), Gambia (13.5%) (28), and Ghana (5.4%) (29). This might be due to time variation and/or due to the increment of awareness among diabetic patients because of the rise of renal failure currently.

### Factors Associated With Knowledge of DM Complications

Governmental workers [AOR = 3.13, 95% CI (0.67, 14.65)] and merchants [AOR = 2.54, 95% CI (1.16, 5.56)] were three times and two times more likely to have good knowledge about DM complications compared to farmers, respectively. Patients who are more than 5 years since diagnosis of DM were less likely to have good knowledge of DM complications. This might be due to those patients who had nearly been diagnosed with DM might give high attention to diabetic educations and counseling.

Those patients who had a family history of DM [AOR = 1.68, 95% CI (1.03, 2.75)] were more likely to have knowledge of diabetic complications. The finding from Northwest Ethiopia also revealed a similar result although it had given a stronger association than (AOR = 5.55) this finding (11). Additionally, patients who had participated in diabetic counseling [AOR = 2.41, 95% CI (1.5, 3.86)] were two times more likely to have good knowledge of diabetic complications.

#### Strength and Limitation of the Study

##### Strength

- The previous study included diabetic patients in one hospital but in this study patients in three hospitals were included.
- This study tried to see the effect of diabetic educations and participation in diabetic counseling on knowledge of DM complications.

##### Limitation

- Lack of references because many research studies were descriptive cross-section studies.
- Since the study design used was cross-sectional, the finding regard to factors associated with knowledge of DM complications might not be strong.

## Conclusion and Recommendation

### Conclusion

Knowledge of diabetic complications among patients with type-2 DM in Public Hospitals of Addis Ababa was 54.9% [95% CI (49.6, 59.5)]. The knowledge of diabetic complications was determined by the duration of DM since diagnosed, current profession, family history, and participation in diabetic counseling.

### Recommendations

For DM clinics and DM federations

- Strengthen the participation of diabetic patients in diabetic counseling
- Emphasize sustaining knowledge about DM complications for patients who are more than 5 years since diagnosed with DM
- Attention is needed for farmers who had DM in increasing their knowledge about DM complications

For patients with DM and healthcare workers

- Patients without family history should increase their knowledge/awareness about DM complications

For researchers

- Researchers are recommended to dig out reasons for knowledge about DM complications to be lower for diabetic patients of long duration by qualitative study design.

## Data Availability Statement

The raw data supporting the conclusions of this article will be made available by the authors, without undue reservation.

## Ethics Statement

The studies involving human participants were reviewed and approved by the Institutional Research Ethics Review Committee of Kea-med Medical College which was written on October 2020 with Ref. No of KMC/IRB/014/12. The patients/participants provided their written informed consent to participate in this study.

## Author Contributions

All authors made a significant contribution to the work reported, whether that is in the conception, study design, execution, acquisition of data, analysis, and interpretation. They contributed to all these areas, taking part in drafting, revising, or critically reviewing the article, giving final approval of the version to be published, agreeing on the journal to which the article has been submitted, and agreeing to be accountable for all aspects of the work.

## Conflict of Interest

The authors declare that the research was conducted in the absence of any commercial or financial relationships that could be construed as a potential conflict of interest.

## Publisher's Note

All claims expressed in this article are solely those of the authors and do not necessarily represent those of their affiliated organizations, or those of the publisher, the editors and the reviewers. Any product that may be evaluated in this article, or claim that may be made by its manufacturer, is not guaranteed or endorsed by the publisher.

## References

[1] Kinaan M Ding H Triggle CR. Metformin: an old drug for the treatment of diabetes but a new drug for the protection of the endothelium. Med Principles Prac. (2015) 24:401–15. 10.1159/000381643 26021280 PMC5588255

[2] American Diabetes Association. Diagnosis and classification of diabetes mellitus. Diabetes Care. (2014) 37(Suppl. 1):81–90. 10.2337/dc14-S081 24357215

[3] International Diabetes Federation. Idf Diabetes Atlas. 9th ed (2019). Available online at: https://www.diabetesatlas.org/upload/resources/2019/IDF_Atlas_9th_Edition_2019.pdf (accessed May 15, 2020).

[4] World Federation for Mental Health. Depression: A Global Crisis; World Mental Health Day, October 10, 2012. (2012). Available online at: https://www.who.int/mental_health/management/depression/wfmh_paper_depression_wmhd_2012.pdf (accessed May 15, 2020).

[5] Guariguata L Whiting D Weil C Unwin N. The International Diabetes Federation Diabetes Atlas methodology for estimating global and national prevalence of diabetes in adults. Diabetes Res Clin Pract. (2011) 94:322–32. 10.1016/j.diabres.2011.10.040 22100977

[6] Feleke SA Alemayehu CM Adane HT. Assessment of the level and associated factors with knowledge and practice of Diabetes Mellitus among Diabetic Patients attending at FelegeHiwot Hospital, Northwest Ethiopia. Clin Med Res. (2013) 2:110–20. 10.11648/j.cmr.20130206.11

[7] Atun R Davies JI Gale EAM Bärnighausen T Beran D Kengne AP. The lancet diabetes & endocrinology commission diabetes in Sub-Saharan Africa: From clinical care to health policy. Lancet. (2017) 8587:1–46. 10.1016/S2213-8587(17)30181-X 28688818

[8] Chowdhury TA Shaho S Moolla A. Complications of diabetes: progress, but significant challenges ahead. Ann Transl Med. (2014) 2:10–3. 10.3978/j.issn.2305-5839.2014.08.12 25568873 PMC4260049

[9] Mao W Yip CW Chen W. Complications of diabetes in China: health system and economic implications. BMC Public Health. (2019) 19:1–11. 10.1186/s12889-019-6569-8 30841928 PMC6414024

[10] Abejew AA Belay AZ Kerie MW. Diabetic complications among adult diabetic patients of a tertiary hospital in northeast Ethiopia. Adv Public Health. (2015) 2015:1–7. 10.1155/2015/290920

[11] Belsti Y Akalu Y Fekadu H Animut Y. Awareness of complications of diabetes mellitus and its associated factors among type 2 diabetic patients at Addis Zemen District Hospital, northwest Ethiopia. BMC Res Notes. (2019) 12:1–7. 10.1186/s13104-019-4637-x 31533856 PMC6751640

[12] Ebru NC Zencir M Fenkc S. Assessment of awareness of diabetic retinopathy and utilization of eye care services among Turkish diabetic patients. Primary Care Diabetes. (2013) 7:297–302. 10.1016/j.pcd.2013.04.002 23639610

[13] Abebe SM Berhane Y Worku A Alemu S. Increasing trends of diabetes mellitus and body weight: a ten year observation at Gondar University Teaching Referral Hospital, Northwest Ethiopia. PLoS ONE. (2013) 8:e60081. 10.1371/journal.pone.0060081 23536904 PMC3607605

[14] Helamo D Delil R Dileba T. Trends of diabetes mellitus and hypertension at Nigist Eleni Mohammed General Hospital, Hossana, Ethiopia (December 2010-January 2014): a five year longitudinal study. Saf Heal. (2017) 3:1–9. 10.1186/s40886-017-0052-y

[15] Omoleke SA. Chronic non-communicable disease as a new epidemic in Africa: focus on the Gambia. Pan Afr Med J. (2013) 8688:1–9. 10.11604/pamj.2013.14.87.1899 23646223 PMC3641923

[16] Gill G V Mbanya J Ramaiya KL Tesfaye S. A sub-Saharan African perspective of diabetes. Diabetologia. (2009) 52:8–16. 10.1007/s00125-008-1167-9 18846363

[17] Reza A Bahreini F Afkhami-Ardekani M. People awareness about diabetes disease and its complications among aged 18 years and older in Bushehr port inhabitants (Iran). Diabetes Metabolic Syndr Clin Res Rev. (2007) 1:3–7. 10.1016/j.dsx.2007.09.003

[18] Paneru N Adhikari RD. Knowledge regarding diabetic complications among diabetic clients attending outpatient department in a tertiary hospital, Kathmandu. J Diabetes Endocrinol. (2019) 10:1–7. 10.5897/JDE2018.0125

[19] Ullah F Afridi AK Rahim F Ashfaq M Khan S Shabbier G. Knowledge of diabetic complications in patients with diabetes mellitus. J Ayub Med Coll Abbottabad. (2015) 27:360–3. Available online at: https://europepmc.org/article/med/26411116 26411116

[20] Fatani EM Gari LN Alharbi AH Ali J. Awareness of diabetic complications, perceived knowledge, compliance to medications and control of diabetes among diabetic population of Makkah City, Kingdome Saudi Arabia: cross-sectional study. Egypt J Hosp Med. (2018) 70:1190–5. 10.12816/0044548

[21] Bodunde OT Odusan O Ogunsemi O Ajibode HA Raimi TH. Awareness of ocular complications of diabetes among diabetic patients in a tertiary hospital in western, nigeria awareness of ocular complications of diabetes among diabetic patients in a Tertiary Hospital in Western, Nigeria. IOSR J Dental Med Sci. (2014) 6:1–5. 10.9790/0853-16620912

[22] Lebeta KR Argaw Z Birhane BW. Prevalence of diabetic complications and its associated factors among diabetes mellitus patients attending diabetes mellitus clinics; institution based cross sectional study. Am J Health Res. (2017) 5:38–43. 10.11648/j.ajhr.20170502.13

[23] Addoor KR Bhandary SV Khanna R Rao LG Lingam KD Binu VS. Assessment of awareness of diabetic retinopathy among the diabetics attending the peripheral diabetic clinics in Melaka, Malaysia. Med J Malaysia. (2011) 66:48–52. Available online at: http://www.e-mjm.org/2011/v66n1/Diabetes_mellitus.pdf 23765143

[24] Year F Students M Lumpur K Lumpur K Khor B. Awareness of eye complications and prevalence of retinopathy in the first visit to eye clinic among type 2 diabetic patients. Int J Ophthalmol. (2005) 4:519–24. 10.3980/j.issn.2222-3959.2011.05.12 22553714 PMC3340716

[25] Franzco JSM Franzco HSN Fafphm PR Ramsay E Aung M Ophth D. Original Article Awareness of diabetic eye disease among general practitioners and diabetic patients in Yangon, Myanmar. Clin Exp Ophthalmol. (2008) 36:265–73. 10.1111/j.1442-9071.2008.01724.x 18412597

[26] Durgad A Parakh RB Dhananjaya M Ramesh KN. Awareness of diabetes mellitus and its complications among patients at tertiary care hospital. Int J Scient Study. (2016) 4:17354. 10.17354/ijss/2016/200

[27] Hoque MA Nazmul HAM. Knowledge of diabetic complications in a diabetic population. J Med. (2009) 10:90–3. 10.3329/jom.v10i2.2821

[28] Foma MA Saidu Y Omoleke SA Jafali J. Awareness of diabetes mellitus among diabetic patients in the Gambia: a strong case for health education and promotion. BMC Public Health. (2013) 13:1–8. 10.1186/1471-2458-13-1124 24304618 PMC3913398

[29] Obirikorang Y Obirikorang C Anto EO Acheampong E Batu EN Stella AD. Knowledge of complications of diabetes mellitus among patients visiting the diabetes clinic at Sampa Government Hospital, Ghana: a descriptive study. BMC Public Health. (2016) 16:1–8. 10.1186/s12889-016-3311-7 27457072 PMC4960830

