# Supplementary material for: Is the Duration of Diabetes Diseases Positively Associated With Knowledge About Diabetic Complications? Knowledge of Diabetes Mellitus Complications and Associated Factors Among Type-2 Diabetic Patients in Public Hospitals of Addis Ababa, 2020
Source: Front Public Health. 2022 Feb 21;9:812586. doi: 10.3389/fpubh.2021.812586 (PMC8899007; doi:10.3389/fpubh.2021.812586)
Supplement: Supplementary file 1 [file Table_1.DOCX]

# APPENDIX

## Questioners

## Appendix 1. Information Sheet

KIAMED MEDICAL COLLEGE

DEPARTMENT OF ADULT HEALTH NURSING

Good morning/afternoon dear participant! My name is _________________. I am working as a data collector for the study being conducted in this institution on Knowledge of Diabetic Complication among Type-2 Diabetes Patients in Public Hospitals of Addis Ababa, Ethiopia by Getachew Zewdu, Daniel Mengistu and Addisu Waleligne. I kindly request you to lend me your attention to explain the study and how being you selected as the study participant.

**Title of the Research Project:** Knowledge of Diabetic Complication among Type-2 Diabetes Patients in Public Hospitals of Addis Ababa, Ethiopia

**Name of the Sponsor**: Self

**Purpose of the Research Project**: The study aims to determine the level of knowledge of diabetes complications and to identify factors affecting knowledge of diabetic complications among type-2 diabetic patients in Public Hospitals of Addis Ababa.

**Procedure:** For this study, three hospitals in Addis Ababa are included. Interviewer-administered questioners are provided to participants and they will answer to questions; then, the data will be collected by the assigned data collectors.

**Risk and Benefits:** The study has no direct benefit for those study participants but they may be indirectly beneficial if the result utilized by planners. Addis Ababa Health Office, each District Health Office, and hospitals will get the final result of the study. There is no risk due to participating in this study.

.

**Confidentiality**: No need of registering your name; therefore, the information you gave will be kept confidential. All information collected will be kept confidential and destroyed two years after the end of the project. No other person besides the research team will see it.

**Right to Refusal or Withdraw**: To start data collection, approval of the individual participant is required. If you are not willing to participate you can refuse.

**Person to contact**: If you have any further questions or would like to receive further information about the project, please contact:

Name: Getachew Zewdu:- Email: [gech.zewdu@gmail.com](mailto:gech.zewdu@gmail.com) (Principal Investigator) Cell Phone: +251 910561191 (Principal Investigator)

Daniel Mengistu:- Email: mengistudaniel[@yahoo.com](mailto:amsalu.alagaw@yahoo.com) (Advisor)

Addisu Waleligne: [addisuwalelign21@gmail.com](mailto:addisuwalelign21@gmail.com)

Thank you for taking the time to give the Information, and asking any questions that you might have had.

Can we proceed?

Yes _______________ No___________________

Participant’s Signature _______________________________

## Appendix 2.Questionnaire

**1. Socio-demographic questions**

|  | Questions | Response | |
| --- | --- | --- | --- |
| 101 | Age (in a year) |  | |
| 102 | Sex | 1. Male 2. Female | |
| 103 | What is your education status? | 1. Cannot write and read 2. Read and write 3. Primary 4. High school and above | |
| 104 | Marital status | 1. Single 2. Married 3. Divorced 4. Widowed | |
| 105 | What is your current profession? | 1. Farmer 2. Governmental worker 3. Merchant 4. Housewife 5. NGO worker | |
| 106 | What is your religion? | 1. Orthodox 2. Muslim 3. Protestant 4. Catholic 5. Other……….. (specify) | |
| 107 | Where is your residence? | 1. Rural 2. Urban | |
| 108 | When did you know that you had DM? | ……………… | |
| 109 | What type of medication do you use? | 1. Oral 2. Injectable 3. Both | |
| 110 | Is there a family member who had DM? | 1. Yes 2. No | |
| 111 | How much is your monthly income in Birr? | 1. <500 2. 500-1500 3. 1501-2500 4. >2500 | |
| 112 | Have you been informed about complication of diabetes? | - 1. Yes   2. No | If the answer is no skip to ques. No. 114 |
| 113 | If the answer fo question 112 is yes, where did you get information about DM complication? | 1. Health worker 2. Friend/parent 3. TV/radio 4. Other^*^ | |
| 114 | Have you ever participated in diabetes counseling | - 1. Yes 2. No | |

**2.** Knowledge on DM complication among type-2 DM at Addis Ababa Hospitals

| 201 | What is the normal fasting blood sugar level? | 1. <70 mg/dl 2. 70-110 mg/dl 3. >126 mg/dl 4. Don’t know |  |
| --- | --- | --- | --- |
| 202 | What are the most common symptoms of high blood sugar? | 1. Increased thirst……… yes/no/ I don’t know 2. Frequent urination…... yes/no/ I don’t know 3. Blurring of vision…… yes/no/ I don’t know 4. Weakness…………… yes/no/ I don’t know 5. Dry mouth…………... yes/no/ I don’t know 6. Confusion …………... yes/no/ I don’t know |  |
| 203 | What are the most common symptoms of low blood sugar? | 1. Palpitation………….. …yes/no/ I don’t know 2. Tremor……………… …yes/no/ I don’t know 3. Sweating…….. …………yes/no/ I don’t know 4. Blurring of vision……….yes/no/ I don’t know 5. Decreased coordination… yes/no/ I don’t know |  |
| 204 | Do you know that diabetes can cause complications in organs of our body? | 1. Yes 2. No | If no , skip to question no 206 |
| 205 | If yes for question no 204, list some organs complications | 1. Diabetic foot…………...yes/no/ I don’t know 2. Eye complication………yes/no/ I don’t know 3. Heart complication……..yes/no/ I don’t know 4. Neuropathy……………..yes/no/ I don’t know 5. Renal complications….....yes/no/ I don’t know 6. Stroke…………………....yes/no/ I don’t know 7. Teeth decay…………..….yes/no/ I don’t know 8. Hypertension…………….yes/ no/ I don’t know 9. Sexual dysfunction ……...yes/no/ I don’t know |  |
| 206 | Can dietary modification prevent diabetic complication? | 1. Yes 2. No 3. I don’t know |  |
| 207 | Can Stop smoking/and alcohol stopping prevent diabetic complication? | 1. Yes 2. No 3. 3. I don’t know |  |
| 208 | Is physical work or exercise help to prevent diabetes complication? | 1. Yes 2. No 3. I don’t know |  |
| 209 | If you are beginning to have a low blood glucose reaction, you should? | 1. Exercise………………...yes/no/ I don’t know 2. Lie down and rest………yes/no/ I don’t know 3. Drink some juice……..…yes/no/ I don’t know 4. Take rapid-acting insulin.. yes/no/ I don’t know |  |
| 210 | What should you do when your blood sugar is raised? | 1. Dietary modification…….yes/no/ I don’t know 2. Physical exercise………....yes/no/ I don’t know 3. Lowering stress…………..yes/no/ I don’t know 4. Take insulin…………..….yes/no/ I don’t know |  |
| 211 | Has diabetic education existed in the facility where you had follow-up? | 1. Yes 2. No 3. I don’t know |  |

## Appendix 3

**መጠይቅ**

ኪያሜድ ሜድካል ኮሌጅ

የነርሲንግ ትምህርት ክፍል

መግቢያ: ሰላም ውድ ተሳታፊያችን! ስሜ ………………………………….እባላለሁ፡፡ በኪያሜድ ሜድካል ኮሌጅ ጌታቸው ዘውዱ፣ ዳነኤል መንግስቱ እና አዲሱ ዋለልኝ "በአዲስ አበባ ውስጥ በመንግስት ሆስፒታሎች የሚታከሙ የስኳር በሽታ ህሙማን ከስኳር በሽታ ጋራ ተያይዘው ሰለምያጋጥሙ ውስብስብ የጤና ችግሮች ያላችውን ግናዛቤ መዳሰስ" በሚል ርዕስ ለሚሰሩት ጥናት መረጃ ሰብሳቢ ነኝ፡፡ በመሆኑም አጠቃላይ ስለጥናቱና እርስዎ እንዴት እንደተመረጡ እንዳብራራለዎት በትህትና እጠይቃለው፡፡

**የምርምሩ ርዕስ:** "በአዲስ አበባ ውስጥ በመንግስት ሆስፒታሎች የሚታከሙ የስኳር በሽታ ህሙማን ከስከር ጋራ ተያይዘው ሰለምያጋጥሙ ውስብስብ የጤና ችግሮች ያላችውን ግናዛቤ መዳሰስ"

**ለምርምር ድጋፍ ያደረገው:** በግል

**የጥናቱ ዓላማ:** የዚህ ጥናት ዋና ዓላማ በአዲስ አበባ ውስጥ በመንግስት ሆስፒታሎች የሚታከሙ ስኳር በሽታ ህሙማን ከስኳር በሽታ ጋራ ተያይዘው ሰለምያጋጥሙ ውስብስብ የጤና ችግሮች ያላችውን ግናዛቤ መዳሰስ እና ግንዛቤያቸውን የሚወስኑትን ነገሮች መለየት ነው፡፡

**አተገባበር:** ጥናቱ አዲስ አበባ በሚገኙ ሶስት ሆስፒታሎች ይካሄዳል። መጥይቆቹ በጥናቱ የሚሳተፉ ተሳታፊዎችን በመጠየቅ መልስ እንዲሰጡ ከተደረገ በኋላ መጠይቆቹ ይሰበሰባሉ።

**ጥቅሞና ጉዳት:** በጥናቱ ውስጥ የሚካተቱት የጥናቱ ተሳታፊዎች ምንም ዓይነት ቀጥተኛ ጥቅም አያገኙም። ነገር ግን ጥናቱ ከተጠናቀቀ በኋላ የጥናቱ ውጤትን መሰረት አድርጎ በሚዘጋጁ እቅዶች በተዘዋዋሪ ተጠቃሚ ሊሆኑ ይችላሉ። የአዲስ አበባ ጤና ቢና የሆስፒታል ስራ አስኪያጆች የጥናቱ ውጤት እንዲደርሳቸው ይደረጋል።በጥናቱ ተሳታፊ በመሆንዎ የሚጎዱት ነገር አይኖርም፡፡

**ሚስጥራዊነት:** የጥናቱ ተሳታፊዎች ስማቸውን አይፃፍም፡፡ ስለዚህ የሚሰጡት መረጃው ሚስጥራዊነቱ የተጠበቀ ነው፡፡ መረጃው በጥንቃቄ የሚያዝ ሲሆን ከሁለት ዓመት በኋላ ይቃጠላል፡፡ መብት: የጥናቱ ተሳታፊዎች በጥናቱ ያለመሳተፍ መብት አላቸው፤ እንዲሁም በየትኛው ሰዓት ማቋረጥ ይችላሉ።

ስለምርምሩ ጥያቄ ካለዎት ወይም ተጭማሪ ማብራሪያ ካስፈለገዎት የሚከተሉትን አድራሻዎችን ይጠቀሙ።

ጌታቸው ዘውዱ:- ኢ-ሜይል: [gech.zewdu@gmail.com](mailto:gech.zewdu@gmail.com)

ሞባይል ስልክ:+251910561191 (የዋና ተመራማሪ)

ዳንኤል መንግስቱ (Asst. prof)፡- ኢ-ሜይል: [mengistudaniel@yahoo.com](mailto:mengistudaniel@yahoo.com) (አማካሪ)

አዲሱ ዋለልኝ ፡ ኢሜል፡- [addisuwalelign21@gmail.com](mailto:addisuwalelign21@gmail.com)

ጊዜ ሰጥተው ይህንን የመረጃ ቅጽ ስላነበቡና ስለሚኖረዎ ማንኛውም ጥያቄ በቅድሚያ እናመሰግናለን።

ለቃለ መጠይቁ ፈቃደኛ ነዎት

አዎ ) አይደለሁም)

የተሳታፊ ፊርማ__________________________________________

## Appendix 3.1 የመረጃ መሰብሰቢያ ፎርም

**1.** የማህበራዊና ኢኮኖሚያዊ መረጃዎች (**Socio-demographic questions**

|  | ጥያቄዎች | መልስ |
| --- | --- | --- |
| 101 | እድሜ (በአመት) |  |
| 102 | ጾታ | 1. ወንድ 2. ሴት |
| 103 | አሁን ላይ ያሉበት የትምህርት ደረጃ | 1. ማምበብና መጻፍ የማይችሉ 2. የመጀመሪያ ደረጃ 3. ሁለተኛ ደረጃና ከዚያ በላይ |
| 104 | የጋብቻ ሁኔታ | 1. ያላገባ/ች 2. ያገባ/ች 3. የፈታ/ች 4. ሚስቱ የሞተችበት/ ባሏ የሞተባት |
| 105 | አሁን ላይ የሚሰሩበት የሙያ ዘርፍ ምንድ ነው? | 1. ግብርና 2. የመንግስት ሰራተኛ 3. ነጋዴ 4. የቤት እመቤት 5. መንግስታዊ ያልሆነ ድርጅት |
| 106 | የሚከተሉት እምነት ምንድነው? | 1. ኦርቶዶክስ 2. እስልምና 3. ፕሮቴስታንት 4. ካቶሊክ 5. ሌላ…………….(ይግለጹ) |
| 107 | የሚኖሩት የት አካባቢ ነው? | 1. ገጠር 2. ከተማ |
| 108 | የስኳር በሽታ እንዳለበወት ካወቁ ምን ያህል ጊዜ ሆነዎት? | ……………… |
| 109 | ለስኳር በሽታ ቁጥጥር የሚጠቀሙት ምን አይነት መድሃኒት ነው? | 1. በአፍ የሚወሰድ 2. በመርፌ የሚሰጥ 3. ሁለቱንም |
| 110 | ከርስዎ በፊት በቤተስበዎ ውስጥ የስኳር በሽታ ታማሚ ነበር? | 1. አዎን 2. የለም |
| 111 | በወር ውስጥ የሚያገኙት የገቢ መጠን ምን ያክል ነው? | 1. <500 2. 500-1500 3. 1501-2500 4. >2500 |
| 112 | \| ስለስኳር በሽታ ተያያዥ ውስብስ የጤና ችግሮች መረጃ ሰምተው ያውቃሉ? \| 1. አዎ 2. የለም \| \| --- \| --- \| | መልስዎ የለም ከሆነ ወደ ጥያቄ 114 ይሻገሩ |
| 113 | ለጥያቄ ቁጥር 112 መልስዎ አዎን ከሆነ ስለስኳር በሽታ ተያያዥ ውስብስ የጤና ችግሮች መረጃ ከየት ነው የሰሙ? | 1. 1. ከጤና ባለሙያ 2. 2. ከጋደኛ/ቤተሰብ 3. 3. ከቴሌቪዥን/ራዲዮ 4. 4. ከሌላ………….(ይጥቀሱ) |
| 114 | ስለስኳር በሽታ በሚሰጡ የምክር አገልግሎቶች ተሳተፈው ያውቃሉ | 1. አዎ 2. የለም |

**2.** በአዲስ አበባ ውስጥ በመንግስት ሆስፒታሎች የሚታከሙ የስኳር ህሙማን ከስከር ጋራ ተያይዘው ሰለምያጋጥሙ ውስብስብ የጤና ችግሮች ያላችውን ግናዛቤ

| 201 | የተለመደው የጾም(ከምግብ በፊት) የደም ስኳር መጠን ምንድነው? | 1. <70 mg/dl 2. 70-110 mg/dl 3. >126 mg/dl 4. አላውቅም | |  |
| --- | --- | --- | --- | --- |
| 202 | ከፍተኛ የደም ስኳር በሽታ መጨመር የተለመዱ ምልክቶች ምንድናቸው? | 1. ጥማት ይጨምራል….አዎ/አይደለም/አላወቅም 2. ተደጋጋሚ ሽንት………አዎ/አይደለም/አላወቅም 3. የማየት ብዥታ………..አዎ/አይደለም/አላወቅም 4. ድክመት………………..አዎ/አይደለም/አላወቅም 5. የአፍ መድረቅ………..አዎ/አይደለም/አላወቅም 6. ግራ መጋባት/የትኩረት መቀነስ አዎ/አይደለም/አላወቅ | |  |
| 203 | በጣም የተለመዱ በደም ውስጥ የስካር መጠን መቀነስ ምልክቶች ምን ምን ናቸው? | 1. የልብ ምት መጨመር…አዎ/አይደለም/አላወቅም 2. መነቀጥቀጥ………………አዎ/አይደለም/አላወቅም 3. ማላብ………………………አዎ/አይደለም/አላወቅም 4. የእይታ ብዥታ…………..አዎ/አይደለም/አላወቅም 5. የተቀነሰ ቅንጅት………..አዎ/አይደለም/አላወቅም | |  |
| 204 | የስኳር በሽታ ለተለያዩ የሰውነት ክፍሎችን ችግር ሊያጋልጥ እንደሚችል ያውቃሉን | 1. አዎ 2. አይደለም | መልስዎ አዎን ካልሆነ ወደ ጥያቄ ቁጥር 206 ይሻገሩ | |
| 205 | ለጥያቄ ቁጥር 204 መልስዎ አዎን ከሆነ ከስኳር በሽታ ጋር ተያይዞ ከሚመጡ ችግሮች መካከል የትኛው ሊከሰት ይችላል? | 1. የእግር ቁስለት….አዎ/አይደለም/አላወቅም 2. የአይን ህመም….አዎ/አይደለም/አላወቅም 3. የልብ ህመም……አዎ/አይደለም/አላወቅም 4. የነርቭ በሽታ……አዎ/አይደለም/አላወቅም 5. የኩላሊት ህመም…አዎ/አይደለም/አላወቅም 6. የአንጎል ህመም…..አዎ/አይደለም/አላወቅም 7. የጥርስ መበስበስ….አዎ/አይደለም/አላወቅም 8. የደም ግፊት………..አዎ/አይደለም/አላወቅም 9. ስንፈተ ወሲብ……..አዎ/አይደለም/አላወቅም | |  |
| 206 | የአመጋገብ ለውጥ የስኳር በሽታ በሽታዎችን መከላከል ይችላል? | 1. አዎ 2. አይደለም 3. አላውቅም | |  |
| 207 | ማጨስ / እና አልኮሆል ማቆም የስኳር በሽታ ችግርን መከላከል ይችላል? | 1. አዎ 2. አይደለም 3. አላውቅም | |  |
| 208 | የስኳር በሽታ ችግርን ለመከላከል አካላዊ ሥራ ወይም የአካል ብቃት እንቅስቃሴ ድጋፍ ነውን? | 1. አዎ 2. አይደለም 3. አላውቅም | |  |
| 209 | ዝቅተኛ የደም ግሉኮስ ምላሽ እየሰጡ ከሆነ ምን ማድረግ ይኖርብዎታል? | 1. የአካል ብቃት እንቅስቃሴ….አዎ/አይደለም/አላወቅም 2. መተኛት/እረፍት ማድረግ…..አዎ/አይደለም/አላወቅም 3. ጥቂት ጭማቂ ይጠጡ……..አዎ/አይደለም/አላወቅም 4. በፍጥነት የሚሰራ ኢንሱሊን ይውሰዱ…..አዎ/አይደለም/አላወቅም | |  |
| 210 | የደም ስኳርዎ ሲጨምር ምን ማድረግ አለብዎት? | 1. የአመጋገብ ስርዓት ማሻሻያ…አዎ/አይደለም/አላወቅም 2. የአካል ብቃት እንቅስቃሴ……..አዎ/አይደለም/አላወቅም 3. ውጥረትን መቀነስ………………አዎ/አይደለም/አላወቅም 4. ኢንሱሊን ውሰድ………………..አዎ/አይደለም/አላወቅም | |  |
| 211 | በሚከታተሉበት ሆሰፒታል ውስጥ የስኳር በሽታ የትምህርት ፕሮግራም እንዳለ ያውቃሉ | 1. አዎ 2. የለም 3. አላውቅም | |  |
